# Fast adaptation of physics-informed hybrid models for pneumatic artificial muscles

**DOI:** 10.3389/frobt.2026.1769141

**Published:** 2026-05-18

**Authors:** Genmeng Wang, Remi Chalard, Jenny Cifuentes, Minh Tu Pham

**Affiliations:** 1 University Lyon, INSA Lyon, Université Claude Bernard Lyon 1, Ecole Centrale de Lyon, CNRS, Ampère, UMR5005, Villeurbanne, France; 2 Laboratory IBISC, Evry University, Paris-Saclay, Evry Cedex, France; 3 ICADE, Department of Quantitative Methods, Faculty of Economics and Business Administration, and the Institute for Research in Technology (IIT), ICAI School of Engineering, Universidad Pontificia Comillas, Madrid, Spain

**Keywords:** fine-tuning, foundation model, model adaptation, physics-informed neural networks, pneumatic artificial muscles, transfer learning

## Abstract

Recent advances in foundation models and physics-informed neural networks have demonstrated remarkable generalization and adaptation capabilities across diverse domains. Inspired by these properties, we investigate the adaptation potential of a previously proposed physics-informed hybrid model (PIHM) designed for pneumatic artificial muscles (PAMs). Through a series of experiments, it is demonstrated that, by incorporating an adapter based on physical prior knowledge, the PIHM model can be fine-tuned to transfer across different entity types while significantly reducing training time and maintaining competitive accuracy. The optimization efficiency of the proposed adapter has also been validated through comparison with other transfer learning techniques, such as full fine-tuning (FFT), partial fine-tuning (PFT), and low-rank adaptation (LoRA). These results suggest that embedding structured prior knowledge within hybrid architectures offers a promising solution for fast adaptation of PIHMs in dynamic system modeling.

## 1 Introduction

Since the Center for Research on Foundation Models (CRFMs) in the Human-Centered Artificial Intelligence (HAI) Institute of Stanford University coined the term “foundation model” in August 2021 (Bommasani et al., 2021), a growing body of research has been conducted on foundation models in various domains, including natural language processing and robotics manipulation. The well-known foundation models, such as BERT (Devlin et al., 2019) and GPT-4 (Achiam et al., 2023) in language processing, DALL-E (Ramesh et al., 2021) in image generation, and RT-X and $\pi_{0.5}$ vision–language–action (VLA) models in robotics, have two common features: they are trained on broad data and can be adapted to a wide range of tasks.

The aim of the foundation model approach is to develop a general model that captures some “knowledge” based on large-scale, sometimes multi-modal, information. The “knowledge” learned from the source tasks is expected to be further adapted to downstream tasks of interest without the need for training from scratch (Zhou et al., 2024; Brohan et al., 2023; Bommasani et al., 2021). The adaptation of foundation models can be achieved through different methods, such as full fine-tuning (FFT), partial fine-tuning (PFT), and parameter-efficient fine-tuning (PEFT) (Houlsby et al., 2019; He et al., 2022; Lv et al., 2023; Wang L. et al., 2025).

A similar trend toward model reuse and efficient adaptation has emerged in the field of physics-informed neural networks (PINNs). PINNs provide a promising framework for solving partial differential equations (PDEs) by integrating neural networks with governing physical laws (Raissi et al., 2017). Alongside their development, numerous studies have explored strategies to adapt pre-trained PINNs to new PDE configurations, aiming to avoid costly re-optimization when system parameters or boundary conditions change (Desai et al., 2021; Prantikos et al., 2023; Wang Y. et al., 2025). In contrast to foundation models, which emphasize large-scale pre-training on massive datasets to achieve broad generality, adaptation in PINNs targets data-efficient specialization, exploiting embedded physical priors to rapidly adjust to closely related problems using limited new information (Torres and Niepert, 2024).

Based on evidence from both foundation models and PINNs that pre-learned representations can be effectively transferred across domains, platforms, and tasks, we examine the adaptation capability of a previously proposed physics-informed hybrid model (PIHM) for pneumatic artificial muscle (PAM) modeling. This hybrid model, proposed by the same authors in 2024 for modeling the quasi-static and thermodynamic behavior of PAM, has shown promising performance compared to traditional analytical models and pure data-driven models, such as feedforward neural networks and recurrent neural networks. More importantly, due to the presence of physical prior knowledge, this hybrid model has also demonstrated good generalization to unseen scenarios within the target system (Wang et al., 2025a; b). In this study, previous findings are extended by probing the adaptation capacity of the same hybrid model to new entity types. An adapter based on prior physics knowledge is introduced to augment the parameter space of a pre-learned PIHM for fine-tuning. The objective is to evaluate whether the underlying structure and physical guidance of the hybrid model facilitate fast adaptation to new dynamic systems, thus aligning with the broader goals of generalization and sample efficiency associated with foundation models.

The remainder of this article is organized as follows: Section 2 provides a review of related work on foundation models, hybrid approaches, and the modeling of PAMs. Section 3 thoroughly describes the proposed algorithm for fast adaptation. Section 4 presents the preparation of the dataset and the experimental setup. This section also presents the results obtained by applying the fast adaptation algorithm to two entity types. Finally, Section 5 summarizes the key findings and discusses potential future research directions.

## 2 Preliminaries/backgrounds/related work

### 2.1 Pneumatic artificial muscles

McKibben PAMs are bio-inspired actuators designed to mimic the function of human skeletal muscles. Due to their muscle-like behavior, along with higher power-to-weight and power-to-volume ratios than those of electric and hydraulic actuators, PAMs are ideal for the development of rehabilitation devices and bio-robotics (Noritsugu and Tanaka, 1997; Park et al., 2014; Ding et al., 2008; Yaxi and Xu, 2021; Kalita et al., 2022).

There are several models in existence for the analysis or control of PAMs, including static and thermodynamic models. A quasi-static model of PAMs focuses on capturing the relationship between the actuator’s internal pressure P , length variation ε , and the generated axial force F . The development of the quasi-static model usually starts by assuming that the PAM is a perfect cylinder and the thread length b remains constant during the contraction. Then, the equilibrium of virtual work W done by the inner pressure P and external force F is denoted as shown in Equations 1–3:

$$dW_{in}=dW_{\mathrm{out}},$$ (1)

$$dW_{in}=P\;dV,\;dW_{\mathrm{out}}=-FdL,$$ (2)

P dV=−FdL, (3)

where dL is the change in length and dV is the change in volume. Researchers have incorporated different estimations of dV/dL into Equation 3 to develop their respective quasi-static model (Chou and Hannaford, 1996; Tondu and Lopez, 2000; Bou Saba et al., 2016). Moreover, a low-order polynomial approximation of dV/dL was employed by Hildebrandt et al. (2003).

The development of a thermodynamic model typically involves the application of the first law of thermodynamics to the gas chamber, relating mass flows ${\dot{m}}_{in}$ and ${\dot{m}}_{\mathrm{out}}$ . To streamline the modeling process, a number of simplifying assumptions are usually employed: (i) the gas is considered to behave as an ideal gas, (ii) the pressure and temperature are assumed to be uniformly distributed within the chamber, (iii) kinetic and potential energy contributions are deemed negligible, (iv) the process is considered adiabatic, and (v) the incoming flow is already at the temperature of the gas in the chamber. In view of these conditions, the time derivative of the internal pressure $\dot{P}$ within the PAM is hereby defined as follows (Equation 4):

$$\dot{P}=\frac{kRT}{V}\left({\dot{m}}_{in}-{\dot{m}}_{\mathrm{out}}\right)-k\frac{\dot{V}}{V}P,$$ (4)

where k=1.4 is the specific heat ratio of air, R=287J/(kg⋅K) is the gas constant for dry air, and T=293K is the temperature. This model can be rewritten by inverting the process and adding the mass flow $\dot{m}={\dot{m}}_{in}-{\dot{m}}_{\mathrm{out}}$ (Equation 5):

$$\dot{m}=\frac{\dot{P}V}{\gamma RT}+\frac{P\dot{V}}{RT}.$$ (5)

This simplified thermodynamic model, first introduced by Richer and Hurmuzlu (2000), has been widely adopted in research related to PAMs.

It is noteworthy that the key point of the modeling, in both the static and thermodynamic models, is the representation of the volume of the PAM. In various studies, the representation of the PAM volume is either based on a geometrical model (Chou and Hannaford, 1996; Tondu and Lopez, 2000; Bou Saba et al., 2016) or approximated using a polynomial (Itto and Kogiso, 2011; Hošovský et al., 2015; Hildebrandt et al., 2003).

Nevertheless, the strong nonlinear and hysteretic behavior exhibited by PAMs indicates that the accuracy of existing models can still be enhanced. The previously proposed PIHM approach learns to estimate the volume and the rate of change of volume of PAMs from data, with the guidance of an analytical model (Equation 3 or Equation 5), and has shown encouraging results in PAM modeling.

### 2.2 Physics-informed hybrid model

In this study, the term “hybrid” refers to the integration of an NN model with an analytical model. A number of hybridization methods have been proposed in the literature, and each method makes use of the strengths of both methodologies (Raissi et al., 2017; Lutter et al., 2019; Hošovský et al., 2015; Gaskin et al., 2023). Among the existing strategies, an approach tailored to the specific challenges of PAM modeling has been developed following the physics-informed paradigms to address their quasi-static and thermodynamic behaviors. This model had been previously proposed by the authors in earlier studies and is now revisited in light of its promising adaptation capabilities and performance across unseen conditions (Wang et al., 2025a; b).

This PIHM of PAMs is designed to estimate the gas chamber volume V from a set of physical input variables. Unlike traditional PINN methods, whose optimization relies on a loss function comprising both physical and data components, the PIHM approach requires only the physical component of the loss function, rendering it more feasible for practical applications. This hybrid model provides good approximations of V and $\dot{V}$ with the guidance of an analytical model and also demonstrates good generalization to unseen trajectories of the same system. Specifically, the neural network receives $x=\left[\varepsilon ,\dot{\varepsilon },P,\dot{P}\right]$ as input, and subsequently, an analytical model as shown in Equation 3 or Equation 5 handles the estimated volume $\hat{V}$ , as well as its partial derivative with respect to the input contraction ratio ε and inner pressure P to form the final prediction $y=\hat{\dot{m}}$ or $y=\hat{F}$ . One can develop a reliable solution for modeling of PAMs following Algorithm 1. It is worth noting that steps 8–12 correspond to the Adam optimizer, a widely adopted stochastic gradient-based optimization algorithm designed for deep neural networks (Kingma and Ba, 2014).

Algorithm 1 Training procedure of physics-informed hybrid model of PAMs.

**Require:** Dataset $D={\left\{x^{\left(n\right)},\;y^{\left(n\right)}\right\}}_{n=1}^{N}$ , hyperparameters, Thermodyna

Initialize neural network parameters θ

**for all** *epochs* **do**
**for all** $\left(x_{k},\;y_{k}\right),k\leftarrow$ 1:K **do**⊳ $k^{th}$ batch from D

${\hat{V}}_{k}\leftarrow \phi \left(x_{k};\;\theta \right)$ ⊳ Apply forward propagation
**if** Thermodyna **then**⊳ Thermodynamic model

${\hat{\dot{V}}}_{k}\leftarrow \frac{\partial {\hat{V}}_{k}}{\partial \varepsilon }{\dot{\varepsilon }}_{k}+\frac{\partial {\hat{V}}_{k}}{\partial P_{k}}{\dot{P}}_{k}$ ⊳ Compute derivative of ${\hat{V}}_{k}$ using automatic differentiation

${\hat{y}}_{k}\leftarrow \frac{{\dot{P}}_{k}{\hat{V}}_{k}}{\gamma RT}+\frac{P_{k}{\hat{\dot{V}}}_{k}}{RT}$ ⊳ Predict mass flow with analytical model
**else**⊳ Quasi-Static Model

$\frac{d{\hat{V}}_{k}}{dL}\leftarrow \frac{\partial {\hat{V}}_{k}}{\partial \varepsilon L_{0}}$ ⊳ Compute derivative of ${\hat{V}}_{k}$ using automatic differentiation

${\hat{y}}_{k}\leftarrow P_{k}\frac{d{\hat{V}}_{k}}{dL}$ ⊳ Predict force with the analytical model
**end** **if**

$L_{k}\leftarrow MSE\left(y_{k},{\hat{y}}_{k}\right)+\frac{\lambda }{2}\|\theta {\|}_{2}^{2}$ ⊳ Compute loss

$g_{k}\leftarrow \nabla_{\theta }L_{k}+\lambda \theta_{k-1}$ ⊳ Compute gradient

$M_{k}\leftarrow \beta_{1}M_{k-1}+\left(1-\beta_{1}\right)g_{k}$ ⊳ Update biased first moment

$v_{k}\leftarrow \beta_{2}v_{k-1}+\left(1-\beta_{2}\right)g_{k}^{2}$ ⊳ Update biased second moment

${\hat{M}}_{k}\leftarrow \frac{M_{k}}{1-\beta_{1}^{k}},\;{\hat{v}}_{k}\leftarrow \frac{v_{k}}{1-\beta_{2}^{k}}$ ⊳ Correct bias

$\theta_{k}\leftarrow \theta_{k-1}-\eta \frac{{\hat{M}}_{k}}{\sqrt{{\hat{v}}_{k}}+\epsilon }$ ⊳ Update parameters
**end for**
**end for**
**return** trained parameters $\theta^{*}$

### 2.3 Adaptation in PINNs

Adaptation in PINNs refers to the reuse of pre-trained models to efficiently solve new but related physical problems, such as PDEs with modified parameters and boundary conditions. Compared to retraining a PINN from scratch, the aim of the adaptation methods is to leverage previously learned solution structures and embedded physical priors to accelerate convergence and reduce computational costs.

Interest in this topic stems from the scalability limitations of traditional PINNs: despite their strong physical consistency, even minor modifications to the problem specification often require complete re-optimization. This constraint has driven the development of adaptation strategies to enhance data efficiency, thereby enabling PINNs to be redeployed to new PDEs with fewer data and computational demands.

To achieve adaptation and transfer learning in PINNs, several techniques have been proposed. Full fine-tuning involves using a pre-trained PINN from a source problem as the base model, followed by optimizing all network parameters for the target problem. Although this approach is effective for closely related tasks, it remains computationally intensive (Penwarden et al., 2023). Partial or lightweight fine-tuning freezes a subset of pre-trained parameters, updating only selected layers to reduce training costs while preserving transferable physical representations (Chakraborty, 2021). Recently, parameter-efficient adaptation methods such as low-rank adaptation (LoRA) have been introduced to the PINN domain (Hu et al., 2021; Majumdar et al., 2023). These techniques inject a small number of trainable parameters into the frozen pre-trained network, enabling rapid and stable adaptation while preserving the original model architecture.

## 3 Fast adaptation of physics-informed hybrid models of PAMs

A key property of PIHM is that its learning process is guided by an analytical model; hence, the learned dynamics are derived from the physics prior. In this study, we focus on transferring the learned dynamics to unseen PAM by adding an adapter, which is also based on physics priors and therefore reduces the time and data needed when modeling new PAMs.

In this section, an adapter Q is introduced as a physical principle-based tuning mechanism for fast adaptation of a pre-trained PIHM. Given a pre-trained PIHM on a target PAM $\Sigma_{0}$ , we can obtain Equation 6:

$$y=f_{\Sigma_{0}}\left(x;\theta^{*}\right),$$ (6)

where $x=\left[\varepsilon ,\dot{\varepsilon },P,\dot{P}\right]$ , $y=\hat{\dot{m}}$ for thermodynamic modeling, $y=\hat{F}$ for quasi-static modeling, and $\hat{\cdot }$ refers to estimation. The pre-trained PIHM consists of two parts: the NN part for predicting V , and the analytical part ensuring that learned parameters are consistent with the laws of physics. This physical adapter Q is designed to encode additional physical prior knowledge into pre-trained models, enabling a fast adaptation to new systems. This proposed method augments the learned PIHM parameter solely by one dimension, where physical priors can be stored as empirical guidance (Equation 7):

$$\tilde{\Theta }=\left\{\left(\theta ,Q\right)\;|\;\theta \in \Theta ,\;\Theta \subset R^{d},\;Q\in R\right\}.$$ (7)

**FIGURE 1:**
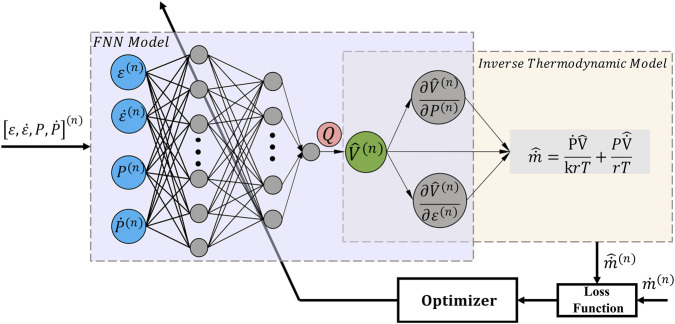
Proposed augmented PIHM for thermodynamic modeling of PAMs.

In scenarios where fast adaptation of $f_{\Sigma_{0}}$ to the new target PAM system $\Sigma_{1}$ is required and the dataset from $\Sigma_{1}$ is small, the original parameter space of the network parameters Θ is proposed to be extended to $\tilde{\Theta }$ by adding an adapter Q , which may also be considered a scalar multiplier. As illustrated in Figure 1, it is proposed to modify the pre-trained PIHM by adding an adapter Q to its output layer. The initial guess of Q can be chosen by roughly dividing the initial volume $V_{1}$ of $\Sigma_{1}$ by the initial volume $V_{0}$ of $\Sigma_{0}$ using the formula for the volume of a cylindrical gas chamber $V=\frac{\pi D^{2}L}{4}$ .

Algorithm 2 outlines the step-by-step implementation of the proposed adaptation method. By providing a dataset $D_{FA}$ , which is much smaller than D required in Algorithm 1, one can follow the steps in Algorithm 2 to fine-tune a pre-trained PIHM to adapt to a different PAM $\Sigma_{1}$ . The tuning process is performed on all pre-trained parameters, and the initially guessed Q with different learning rates as clarified in line 19, Algorithm 2. The learning rate for Q is scaled by a factor of 0.2 compared to the reused neural network parameters, aiming to focus fine-tuning efforts on pre-trained parameters rather than adapters—as the primary behavioral patterns are assumed to be embodied by the pre-trained parameters.

Algorithm 2 Fast adaptation of physics-informed hybrid model of PAMs.

**Require:**Dataset $D_{FA}={\left\{x^{\left(n\right)},\;y^{\left(n\right)}\right\}}_{n=1}^{N_{FA}\ll N}$ , trained parameters $\theta^{*}$ , hyperparameters, Thermodyna

Initial guess of Q ⊳ Based on physical prior

$\tilde{\theta }\leftarrow \left(\theta ,\;Q\right)$ ⊳ Augmented parameters

$\tilde{\phi }\left(x;\;\tilde{\theta }\right)\leftarrow Q\cdot \phi \left(x;\;\theta^{*}\right)$ ⊳ Transfer the pre-learned knowledge
**for all** *epochs* **do**
**for all** $\left(x_{k},\;y_{k}\right),k\leftarrow$ 1:K **do**⊳ $k^{th}$ batch from $D_{FA}$

${\hat{V}}_{k}\leftarrow \tilde{\phi }\left(x_{k};\;\tilde{\theta }\right)$ ⊳ Apply forward propagation
**if** Thermodyna **then**⊳ Thermodynamic model

${\hat{\dot{V}}}_{k}\leftarrow \frac{\partial {\hat{V}}_{k}}{\partial \varepsilon }{\dot{\varepsilon }}_{k}+\frac{\partial {\hat{V}}_{k}}{\partial P_{k}}{\dot{P}}_{k}$ ⊳ Compute derivative of ${\hat{V}}_{k}$ using automatic differentiation

${\hat{y}}_{k}\leftarrow \frac{{\dot{P}}_{k}{\hat{V}}_{k}}{\gamma RT}+\frac{P_{k}{\hat{\dot{V}}}_{k}}{RT}$ ⊳ Predict mass flow with analytical model
**else**⊳ Quasi-static model

$\frac{d{\hat{V}}_{k}}{dL}\leftarrow \frac{\partial {\hat{V}}_{k}}{\partial \varepsilon L_{0}}$ ⊳ Compute derivative of ${\hat{V}}_{k}$ using automatic differentiation

${\hat{y}}_{k}\leftarrow P_{k}\frac{d{\hat{V}}_{k}}{dL}$ ⊳ Predict force with the analytical model
**end if**

$L_{k}\leftarrow MSE\left(y_{k},{\hat{y}}_{k}\right)+\frac{\lambda }{2}\|\tilde{\theta }{\|}_{2}^{2}$ ⊳ Compute loss

$g_{k}\leftarrow \nabla_{\tilde{\theta }}L_{k}+\lambda {\tilde{\theta }}_{k-1}$ ⊳ Compute gradient

$M_{k}\leftarrow \beta_{1}M_{k-1}+\left(1-\beta_{1}\right)g_{k}$ ⊳ Update biased first moment

$v_{k}\leftarrow \beta_{2}v_{k-1}+\left(1-\beta_{2}\right)g_{k}^{2}$ ⊳ Update biased second moment

${\hat{M}}_{k}\leftarrow \frac{M_{k}}{1-\beta_{1}^{k}},\;{\hat{v}}_{k}\leftarrow \frac{v_{k}}{1-\beta_{2}^{k}}$ ⊳ Correct bias

$\theta_{k}\leftarrow \theta_{k-1}-\eta \frac{{\hat{M}}_{k}}{\sqrt{{\hat{v}}_{k}}+\epsilon },\;Q_{k}\leftarrow Q_{k-1}-0.2\cdot \eta \frac{{\hat{M}}_{k}}{\sqrt{{\hat{v}}_{k}}+\epsilon }$ ⊳ Update parameters
**end for**
**end for**
**return**trained parameters ${\tilde{\theta }}^{*}$

## 4 Experimental results

**FIGURE 2:**
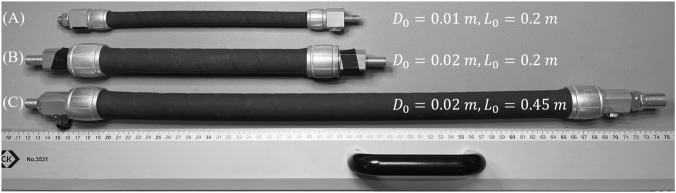
PAM objects in this study: **(A)** PAM with an initial length of 0.2 m and an initial diameter of 0.01 m; **(B)** PAM with an initial length of 0.2 m and an initial diameter of 0.02 m; **(C)** PAM with an initial length of 0.45 m and an initial diameter of 0.02 m.

This section presents the evaluation of the proposed fast adaptation method in thermodynamic modeling of two novel PAM entities, as shown in Figures 2B,C. A PIHM model $\phi_{A}$ is pre-trained with data collected from Figure 2A; then, adapters are added to $\phi_{A}$ for model adaptation, as illustrated in Algorithm 2. The obtained models are noted as $\phi_{B}^{FA}$ and $\phi_{C}^{FA}$ ,where FA stands for fast adaptation. Other transfer learning strategies such as FFT, PFT, and LoRA were also applied in Figures 2B,C to better demonstrate the positioning of the proposed adaptation approach. These adapted models are noted as $\phi_{B}^{\mathrm{FFT}}$ , $\phi_{B}^{\mathrm{PFT}}$ , $\phi_{B}^{\mathrm{LoRA}}$ , $\phi_{C}^{\mathrm{FFT}}$ , $\phi_{C}^{\mathrm{PFT}}$ , and $\phi_{C}^{\mathrm{LoRA}}$ , where the superscripts denote the methods and the subscripts denote the target objects.

**FIGURE 3:**
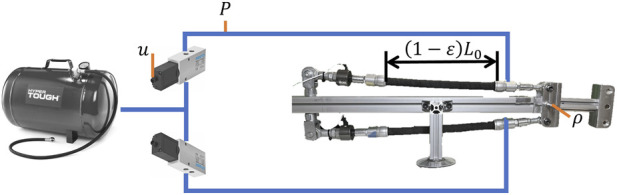
Test bench.

The test bench platform used in the experimental procedure is shown in Figure 3. This platform is mounted on the experimental stand and incorporates a pair of antagonistically connected PAM actuators to enable rotational motion of the right-hand component about its axis. There is also a stable high-pressure gas reservoir capable of supplying 6 bars to both PAMs, with this gas supply controlled using two independent servo valves. The directly measurable values are rotation angle ρ , internal gas pressure P , and the servo valve control voltage u . Furthermore, the mass flow rate $\dot{m}$ can be calculated using the characteristic mapping of the servo valves verified by Olaby et al. (2005). Similarly, the contraction ratio ε can also be determined through a kinematic model of the experimental platform.

### 4.1 Data preparation

Three training datasets $D_{A}$ , $D_{B}$ , and $D_{C}$ were prepared using a simple proportional derivative controller, each containing 20 trajectories sampled at a frequency of 1,000 Hz. These 20 trajectories were all obtained by tracing sinusoidal signals composed of five different frequencies [1, 2, 4, 10] rad/s and five different amplitudes [5, 10, 15, 20, 25]  degrees in rotational axis of the test bench. By switching between different experimental PAM objects, $D_{A}$ , $D_{B}$ , and $D_{C}$ are acquired. By sparsifying the datasets $D_{B}$ and $D_{C}$ , one obtains the datasets $D_{B}^{FA}$ and $D_{C}^{FA}$ for model adaptation. Since the modeling objective of the experimental section is the thermodynamic behavior of the PAM, $x=\left[\varepsilon ,\dot{\varepsilon },P,\dot{P}\right]$ serves as the model input, while $y=\hat{\dot{m}}$ represents the model output. Although both PAMs were actuated during the experiments, only data from the upper muscle were used for model training as the two muscles were identical and demonstrated symmetrical dynamic behavior.

To further investigate whether the adapted PIHM model retains consistent generalization capabilities in unseen scenarios, two validation datasets were prepared for each target system. The first validation dataset is denoted as $D^{\mathrm{tri}}$ , comprising 25 triangular trajectories composed of five frequencies [0.5, 1, 2, 4, 10] rad/s and five amplitudes [5, 10, 15, 20, 25]  degrees . The second validation dataset is denoted as $D^{\mathrm{step}}$ , comprising 10 step trajectories composed of 10 amplitudes [5, 10, 15, 20, 25,−5,−10,−15,−20,−25]  degrees .

### 4.2 Training and fast adaptation

**FIGURE 4:**
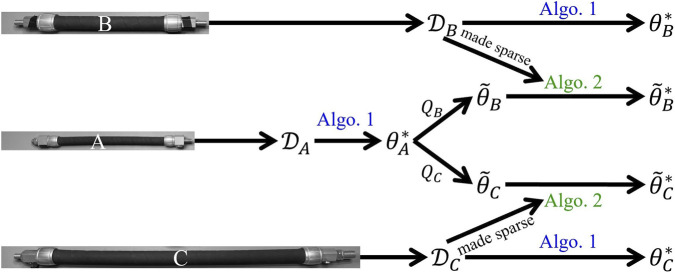
PAM objects in this study: **(A)** the original PAM, a hybrid model was trained from scratch using data collected from this object; **(B)** the first PAM object used to test the proposed fast adaptation algorithm; **(C)** The second PAM object used for fast adaptation.

The workflow of fast adaptation is summarized in Figure 4. A general model was first trained using Algorithm 1 on $D_{A}$ , which is denoted as $\phi_{A}\left(x;\;\theta_{A}^{*}\right)$ . Concurrently, two baseline models were trained from scratch through Algorithm 1 using the $D_{B}$ and $D_{C}$ datasets, denoted as $\phi_{B}\left(x;\;\theta_{B}^{*}\right)$ and $\phi_{C}\left(x;\;\theta_{C}^{*}\right)$ , respectively. The prediction accuracy and training time of these two baseline models are compared with the model obtained using the fast adaptation method, which are $\phi_{B}^{FA}\left(x;\;{\tilde{\theta }}_{B}^{*}\right)$ and $\phi_{C}^{FA}\left(x;\;{\tilde{\theta }}_{C}^{*}\right)$ .

Before executing the fast adaptation of Algorithm 2, initial guesses for $Q_{B}$ and $Q_{C}$ must be drawn from physical knowledge to augment the pre-trained parameters $\theta_{A}^{*}$ into ${\tilde{\theta }}_{B}$ and ${\tilde{\theta }}_{C}$ . As shown in Figure 2, the initial diameter of the air chamber in PAM in Figure 2B is twice that of PAM in Figure 2A, while their initial lengths are identical. The initial diameter of the air chamber in PAM in Figure 2C is twice that of PAM in Figure 2A, and its initial length is 2.25 times that of PAM in Figure 2A. Using the formula for the volume of a cylindrical gas chamber $V=\frac{\pi D^{2}L}{4}$ and considering their initial diameter and length when not in service, one can choose initial guess $Q_{B}=4$ and $Q_{C}=9$ . Based on the initial guesses provided, Algorithm 2 is capable of fine-tuning both the learned parameters $\theta_{A}^{*}$ and the augmented parameters Q to align them with the new PAM system. Furthermore, it is assumed that since the primary behavioral patterns have already been learned through the larger dataset $D_{A}$ , the dataset required for fine-tuning can be sparsified, thereby reducing the time and computational resources needed for knowledge transfer.

The models $\phi_{B}^{\mathrm{FFT}}$ and $\phi_{C}^{\mathrm{FFT}}$ were obtained by fine-tuning all pre-trained parameters in $\phi_{A}$ on downstream datasets $D_{B}$ and $D_{C}$ . On the contrary, only the parameters in the first hidden layer are fine-tuned, while those in the subsequent two layers are frozen when applying the PFT strategy for $\phi_{B}^{\mathrm{PFT}}$ and $\phi_{C}^{\mathrm{PFT}}$ .

The LoRA method injects rank decomposition matrices into layers of a neural network while freezing the pre-trained model weights to reduce the amount of trainable parameters for downstream tasks. In this method, the forward calculation is modified from $h=W_{0}x$ to $h=W_{0}x+BAx$ , where $W_{0}$ is a pre-trained matrix $W_{0}\in R^{d\times k}$ , B is a matrix $B\in R^{d\times r}$ initialized to 0, and A is a randomly initialized matrix $A\in R^{r\times k}$ . By ensuring r≪min(d,k) , one can save both computational time and resources for model adaptation to downstream tasks. In this study, two decomposition matrices $A_{1}\in R^{2\times 128}$ and $B_{1}\in R^{4\times 2}$ were applied to the first hidden layer of $\phi_{A}\left(x;\;\theta_{A}^{*}\right)$ , while $A_{2}\in R^{8\times 128}$ and $B_{2}\in R^{\mathrm{128\times 8}}$ were applied to the second hidden layer. The hyperparameters setup for all aforementioned models can be found in Table 1.

**TABLE 1:** Hyperparameters applied in this study.

Hidden layer	2×128
Activation function	SoftPlus
Learning rate	1e^−4^
Weight decay	1e^−5^
Optimizer	Adam
Loss function	MSE
Number of epochs	10,000
Batch size (training from scratch)	2,048
Batch size (adaptation)	128

### 4.3 Results

A summary of performance metrics can be found in Table 2. The result shows that, with a sparsified dataset and pre-trained parameters, the model adaptation phase requires less time than training from scratch. It is also observed that the models obtained via the proposed fast adaptation approach achieve better prediction precision than the baseline models in the target task, which indicates strong performance in downstream task adaptation. However, on the validation dataset, the model based on the proposed method $\phi^{FA}$ still maintains competitive performance compared to the baseline models ϕ , $\phi^{\mathrm{FFT}}$ , $\phi^{\mathrm{PFT}}$ , and $\phi^{\mathrm{LoRA}}$ , indicating that the proposed adaptation method possesses acceptable generalization capabilities.

**TABLE 2:** Summary of performance metrics.

Target system	(B)					(C)				
Models	Train from scratch	FFT	PFT	LoRA	Ours	Train from scratch	FFT	PFT	LoRA	Ours
# Optimized parameters	17,281	17,281	640	1,288	17,282	17,281	17,281	640	1,288	17,282
L2 -norm in target task	1.17	1.86	1.83	1.40	**1.14**	1.96	2.50	4.68	2.29	**1.65**
L2 -norm in $D^{\mathrm{tri}}$	**1.14**	1.56	1.83	1.43	1.18	1.44	1.50	3.59	**1.44**	2.21
L2 -norm in $D^{\mathrm{step}}$	3.42	**3.38**	3.43	3.61	3.60	**2.47**	2.55	3.46	2.50	2.61
Time used (s)	195,804	469	472	501	471	194,437	470	490	479	463
# training samples	31,464	315				30,952	310			

Values in bold indicate the best results obtained in each task after applying different adaptation methods to each target PAM object.

Under identical computational resources (CPU: Intel i7-1370P, GPU: NVIDIA RTX A500 Laptop), the average time needed for execution of Algorithm 1 is 195,294 s in this study. Meanwhile, the average time required for Algorithm 2 is 467 s, which indicates a significant increase in efficiency. To better appreciate this improvement, it is worth recalling that Algorithm 1 corresponds to full model training from scratch, optimizing the entire parameter set θ , whereas Algorithm 2 performs fast adaptation by optimizing the augmented parameters $\tilde{\theta }$ , starting from the pre-trained model $\phi_{A}\left(x;\theta_{A}^{*}\right)$ . It has been noticed that the LoRA method takes slightly more time during the adaptation phase; this may be due to the need for more computations during the forward prediction process.

**FIGURE 5:**
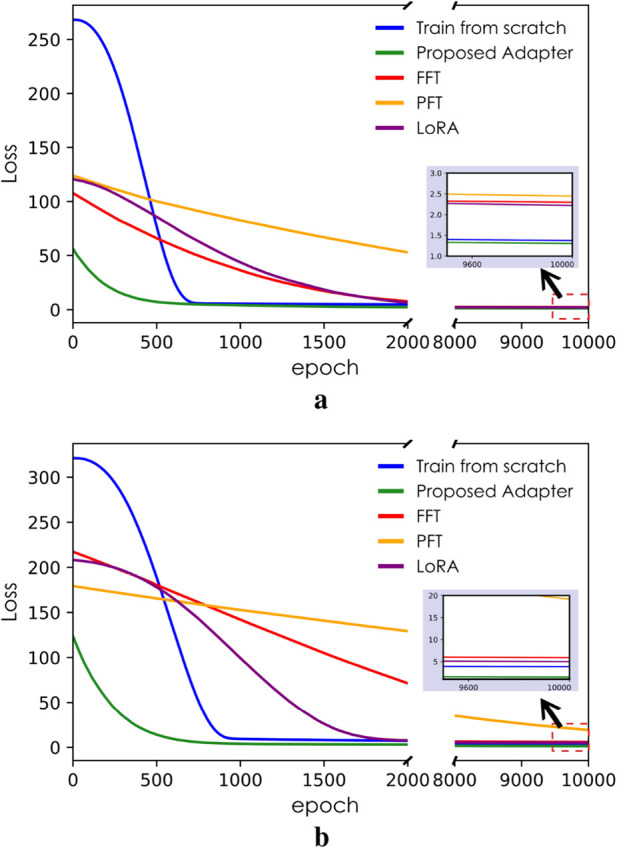
Loss-epoch plot reproduced after training, with the horizontal gap hiding a steady descent phase. **(a)** Training/adaptation executed on dataset $D_{B}$ . **(b)** Training/adaptation executed on dataset $D_{C}$ .

Beyond the enormous savings in computational time, the fast adaptation model also realizes a smaller loss at the end of training. As shown in Figures 5A,B, the loss curve of the proposed method (in green) consistently remains below that of baseline training in the small inset diagram in the right corner. It can also be observed on the left side of Figures 5A,B that the proposed method begins the optimization with a lower initial loss. Since the FFT, PFT, and LoRA methods reuse pre-trained parameters during the adaptation process without any biases in the first prediction, their initial loss during the adaptation phase effectively depends on the substantial knowledge accumulated during the pre-training phase. It can, therefore, be concluded that the proposed adapter helps reduce the initial loss during the adaptation process through additional knowledge based on the initial guess of $Q_{B}$ and $Q_{C}$ . Given that our method converges to the minimum earlier than other adaptation methods, the proposed adaptation strategy has also demonstrated its optimization efficiency. Additionally, it is worth noting that our proposed method does not encounter an initial plateau phase, unlike the training from scratch did at the beginning in Figures 5A,B.

**FIGURE 6:**
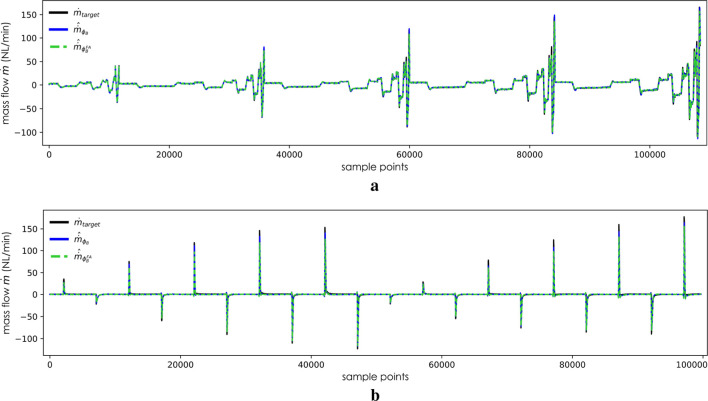
Validation of $\phi_{B}\left(x;\;\theta_{B}^{*}\right)$ and $\phi_{B}^{FA}\left(x;\;{\tilde{\theta }}_{B}^{*}\right)$ in unseen trajectories. **(a)** Predictions by $\phi_{B}\left(x;\;\theta_{B}^{*}\right)$ and $\phi_{B}^{FA}\left(x;\;{\tilde{\theta }}_{B}^{*}\right)$ in $D_{B}^{\mathrm{tri}}$ . **(b)** Predictions by $\phi_{B}\left(x;\;\theta_{B}^{*}\right)$ and $\phi_{B}^{FA}\left(x;\;{\tilde{\theta }}_{B}^{*}\right)$ in $D_{B}^{\mathrm{step}}$ .

**FIGURE 7:**
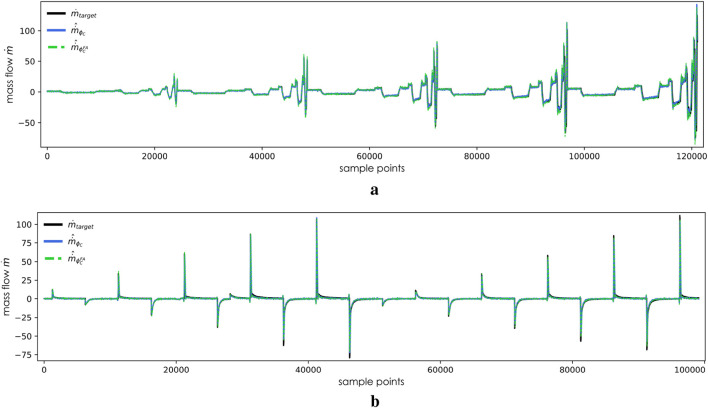
Validation of $\phi_{C}\left(x;\;\theta_{C}^{*}\right)$ and $\phi_{C}^{FA}\left(x;\;{\tilde{\theta }}_{C}^{*}\right)$ in unseen trajectories. **(a)** Predictions by $\phi_{C}\left(x;\;\theta_{C}^{*}\right)$ and $\phi_{C}^{FA}\left(x;\;{\tilde{\theta }}_{C}^{*}\right)$ in $D_{C}^{\mathrm{tri}}$ . **(b)** Predictions by $\phi_{C}\left(x;\;\theta_{C}^{*}\right)$ and $\phi_{C}^{FA}\left(x;\;{\tilde{\theta }}_{C}^{*}\right)$ in $D_{C}^{\mathrm{step}}$ .

On the validation dataset, predictions made by the derived models indicate that despite a relatively long plateau period following rapid descent, none of the four models $\phi_{B}$ , $\phi_{C}$ , $\phi_{B}^{FA}$ , and $\phi_{C}^{FA}$ encountered remarkable overfitting issues. The predictions by the fast adapted models and train from scratch models can be found in Figures 6, 7. The upper subplots of these two figures display results in the validation datasets $D_{B}^{\mathrm{tri}}$ and $D_{C}^{\mathrm{tri}}$ . Correspondingly, the lower subplots present results in the validation datasets $D_{B}^{\mathrm{step}}$ and $D_{C}^{\mathrm{step}}$ .

In Figure 6A, it can be observed that $\phi_{B}$ and $\phi_{B}^{FA}$ deliver nearly identical predictions when sharing the same inputs. Consequently, in Figure 7A, the divergence between the train from the scratch model and the adapted model becomes more pronounced, particularly evident during the negative half-cycle of second-high frequency trajectories. The L2 norm calculated from the validation dataset $D^{\mathrm{tri}}$ in Table 2 supports these findings. The deviation between $\phi_{B}$ and $\phi_{B}^{FA}$ is smaller than the deviation between $\phi_{C}$ and $\phi_{C}^{FA}$ . A possible explanation may be attributed to geometric differences. The increased length of the PAM may result in a smaller-than-expected outflow as the distance between the air at the end of the gas chamber and outlet in the PAM in Figure 6C is longer than that in the PAM in Figure 6A. At relatively lower dynamic frequencies, this deviation is less pronounced because the required predicted gas flow rate is smaller. Similarly, at relatively higher motion frequencies, this phenomenon is not evident, possibly because the tension in the antagonistically connected PAM compensates for it;, hence, the observation is only noted in the trajectory at the second-highest frequency.

The train from scratch models and fast adapted models present similar biases in Figures 6B, 7B, with more noticeable deviations at peak values. The L2 norm calculated in Table 2 indicates that the models perform less favorably on the $D^{\mathrm{step}}$ dataset than on the $D^{\mathrm{tri}}$ dataset. A plausible reason is that the step trajectories in the validation dataset $D^{\mathrm{step}}$ have fewer common features with the training dataset, thereby increasing the difficulty of model prediction. When confronted with step trajectories, $\phi_{B}$ and $\phi_{B}^{FA}$ perform slightly less favorably than $\phi_{C}$ and $\phi_{C}^{FA}$ , as shown in Table 2. This is presumed to the broader prediction range of $D_{B}^{\mathrm{step}}$ than that of $D_{C}^{\mathrm{step}}$ . This difference is illustrated in the Y-axis values between Figures 6B, 7B. A broader prediction range may encompass patterns not covered by $D_{A}$ , thereby increasing the difficulty of the prediction task depicted in Figure 6B.

Despite the performance divergence between ϕ and $\phi^{FA}$ on the validation datasets, this does not imply that the proposed fast adaptation approach is unsatisfactory. On the contrary, it demonstrates that this approach performs better when fine-tuning models to fit specific tasks, such as trajectory data used for fine-tuning.

The fast adapted model was subsequently deployed on a dSPACE MicroLabBox II platform for real-time control. With four times of forward calculations required, the control loop maintained a sampling rate of 1,000 Hz, which is suitable for our application. Moreover, as shown in Table 2, the number of parameters in the fast adapted model is nearly identical to that of the model trained from scratch, indicating nearly equivalent computational complexity. This highlights the advantage of the proposed adaptation method: it neither increases computational complexity nor expands the parameter space beyond a single dimension.

## 5 Conclusion

Based on two key characteristics of foundational models, the authors were motivated to investigate the adaptation capability of their previously suggested physics-informed hybrid model for PAMs. In this regard, a study was conducted by comparing the baseline model with the fast adaptation model in terms of computational time, estimation accuracy, and generalization capability to unseen scenarios. Three types of PAM entities were implemented in the experiments: one for pre-training and two for fine-tuning. The results demonstrate that incorporating human-knowledge-based adapters can significantly reduce computational and data requirements. Furthermore, the fast adaptation model outperformed the baseline models on the fine-tuning dataset but failed to fully match the performance of the baseline model on the validation dataset. This indicates that the model has acquired stronger task specificity, consistent with findings from other studies on foundation model fine-tuning. It is important to emphasize that this work is not intended to develop a foundational model since it was not trained on large-scale data and its generalization capabilities were evaluated on only a limited number of tasks.

These findings are regarded as offering a novel perspective for constructing foundational models for physics-informed representation. In PAM modeling, an intermediate physical quantity—namely, actuator volume—is introduced as a latent representation, while downstream applications may employ distinct analytical models to recover task-specific outputs such as force or airflow. A corresponding modeling approach exists within robotic systems: intermediate representations such as mass matrices serve as shared latent structures for both direct and inverse dynamics formulations. The selected intermediate variable has some relation when it comes across entities. For PAM models, the proposed adapter captures geometric scaling variations between actuators. Similarly, in robotic arm systems, geometric and inertial scaling effects arise due to variations in link lengths, masses, and degrees of freedom. Based on this structural correspondence, the aim of our ongoing study is to develop a more fundamental paradigm for physics-informed robotic arm models. These models utilize training datasets significantly larger than those employed in PAM studies, and the evaluation of the proposed adaptation paradigm can then be conducted across a broader spectrum of robotic scenarios.

## Data Availability

Publicly available datasets were analyzed in this study. These data can be found at https://github.com/GenmengWANG/FastAdaptationOfPIHTM.
